# Two Novel Information Entropy Indices for Analysis of the Eddy Current Distribution

**DOI:** 10.3390/e20090699

**Published:** 2018-09-12

**Authors:** Guolong Chen

**Affiliations:** School of Mechanical and Electrical Engineering, Lanzhou University of Technology, Lanzhou 730050, China; guolongchen@lut.edu.cn

**Keywords:** eddy current testing, nondestructive testing, fractal geometry, information entropy

## Abstract

The Koch curve exciting coil eddy current sensor is a kind of novel flexible planar eddy current probe. In this study, an intersection angle spectrum entropy index and a radial direction energy spectrum entropy were proposed to evaluate the eddy current distribution. Eddy current distributions induced by one turn of a circular coil and one turn of a second order Koch curve coil feed with different exciting frequency alternative currents and at different lift-off distances, were simulated and the eddy current distributions varying with lift-off distance in different exciting frequencies were compared by the two proposed indices. With the increase of the lift-off distance or the decrease of exciting frequency, the similarity between the shape of the Koch curve and the eddy current distribution became weakened and the degree of the concentration of the eddy current distribution in the specimen under the exciting coil was loosened.

## 1. Introduction

Eddy current testing is a type of nondestructive testing method used to detect surface and subsurface defects in conductive materials and their parts. Eddy current testing is vulnerable to the lift-off effect, which is an effect when the distance between the eddy current sensor and the surface of the specimen changes [1]. There are different methods to suppress the effect such as signal processing [2], lookup table [3], sensor design [4,5] and so on. For the sensor design method, the sensors are designed with differential structures to be immune to the effect and there are two kinds of differential eddy current sensors: differential exciting type and differential pickup type [6].

However, the traditional rigid differential eddy current sensors cannot immunize against the lift-off effect when the specimen has a complex surface as the lift-off distance of two differential parts are not equal and the lift-off effect for the two differential parts cannot cancel each other. Thus, flexible eddy current sensors were proposed because its shape changes with the surface of the specimen to minimize the lift-off distance [7]. Up until now, many different coil structures of the flexible eddy current sensors have been designed such as a circular spiral structure [8], meandering winding magnetometer structure [9,10], trapezoidal structure [11], double rectangle structure [12,13], rosette structure [14,15], dual driver structure [16], gradient winding coil structure [17], mesh-winding structure [18], new rosette-like structure [19], same direction line structure [20] and so on. 

The sensitivity of the eddy current sensor is affected by the eddy current distribution. Thus, Chen et al. [21] proposed using the fractal Koch curve as exciting coils of the eddy current sensor. To quantitatively study the eddy current distribution, Zhang et al. [22] and Chen et al. [23] proposed using the angular spectrum and information entropy.

However, the change of the eddy current distribution induced by the Koch curve with a change in the lift-off distance in different exciting frequencies has not been studied. Thus, in this study, two kinds of information entropy indices were proposed to quantitatively research the change of eddy current distribution with the change in the lift-off distance. One index was used to evaluate the similarity between the eddy current distribution and the shape of the exciting coil; another index was used to evaluate the degree of the concentration of eddy current distribution under the exciting coil. Then, eddy current distributions induced by one turn of the second order Koch curve and one turn of the circular coil with different lift-off distances and exciting frequencies were comparatively studied by two proposed information entropy indices.

## 2. Methodology 

The current investigation involved the simulations and analysis of two types of eddy current sensor exciting coils to study the lift-off effect. In one model, the exciting coil was one turn of a second order Koch curve whose circumscribed circle diameter was 10 mm and which was generated by a method in [22]; in the other model, the exciting coil was one turn of a 10 mm diameter circular winding. The eddy current distributions of the different lift-off distances were analyzed by two proposed Shannon information entropy indices. The difference of the two entropy indices were the selections of the discrete random variable and the probability density function.

In the finite element models, the geometric dimension was adjusted as paper [23]. Every model included three parts: a specimen domain, an air domain and an exciting coil. The physical dimensions and properties of the models were identical. The size of the specimen domain and the air domain were 35 mm × 35 mm × 3 mm and 40 mm × 40 mm × 20 mm, respectively. The electrical conductivity, relative permittivity and relative permeability of the specimen were $3.77\times 10^{7}\;S/m$, 1 and 1, respectively; the physical properties of the air domain were 10 S/m, 1 and 1, respectively. The two kinds of exciting coils were arranged above the upper surface of the specimen and the center of each exciting coil overlapped the center axis of the specimen. The distance between the exciting coil plane and the upper surface of the specimen changed from 0.1 mm to 3 mm with a step of 0.1 mm when the lift-off distance was less than 3 mm; the lift-off distance changed from 3 mm to 9 mm with a step of 0.5 mm when the distance was larger than 3 mm. All models were computed in the frequency domain by the AC/DC model in the commercial software COMSOL multi-physics 5.3. The frequencies in the models were 1 KHz, 10 KHz, 20 KHz, 50 KHz, 100 KHz, 200 KHz, 500 KHz, 1000 KHz, 2000 KHz, 5000 KHz and 10,000 KHz. The control function and other setting details were in accordance with [22].

The two analytical methods followed these steps: extract the eddy current vectors, establish random variables, statistics probability distribution function and calculate the Shannon information entropy. 

Then, the eddy current vectors $\left(J_{x},J_{y}\right)$ under the upper surface of specimen 0.01 mm were analyzed; the eddy current vector in a 30 mm×30 mm square plane was extracted by MATLAB 2016a with the step of 0.2 mm in the two directions, so 22,801 points eddy current vectors were extracted at every lift-off distance and every exciting frequency.

Two kinds of Shannon information entropy indices were proposed to analyze the eddy current distribution varying with the lift-off distance in the different exciting frequencies. The first index was proposed to analyze the similarity between an eddy current distribution and an exciting coil, which could be thought of as the information of an exciting coil in its eddy current distribution and is referred to as an intersection angle spectrum entropy (IASE) in the paper. The second index was proposed to analyze the degree of the concentration of the eddy current distribution under the exciting coils and was defined as a radial direction energy spectrum entropy (RDESE).

There were four steps of the IASE index algorithm:

(1) A sample space for IASE was established. In this algorithm, the intersection angle between the perpendicular direction of the position angle at the eddy current extracted point and the direction of the eddy current was used. The angle ranged from 0° to 90°.
(2) Then, the angle range, from 0° to 90°, was equally divided into 18 events with a step of 5° as a discrete random variable.
(3) An intersection angle spectrum $p_{\theta }\left(i\right)$ was created. If the angle of the eddy current vector calculated in step 1 was in the *i*-th event, the energy $J^{2}$ was added in $p_{\theta }\left(i\right)$. Then, the $p_{\theta }\left(i\right)$ was normalized. This step can be expressed as
  (1) $$p_{\theta }\left(i\right)=\frac{\sum J_{5{}^{\circ}\left(i-1\right)<\theta \leq 5{}^{\circ}i}^{2}}{\sum J_{0{}^{\circ}\leq \theta \leq 90{}^{\circ}}^{2}}\;$$
(4) Use the equation of the Shannon information entropy to calculate the index IASE $H_{\theta }$. This step can be expressed as
  (2) $$H_{\theta }=-\overset{18}{\underset{i=1}{\sum }}p_{\theta }\left(i\right)logp_{\theta }\left(i\right)$$

**Figure 1:**
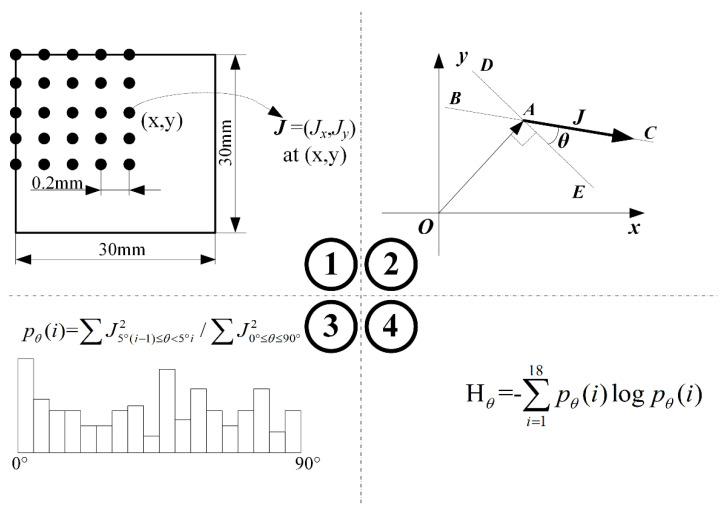
The four steps to calculate the index of the intersection angle spectrum entropy (IASE).

In this paper, the base of the logarithm algorithm was 2, so the units of the IASE and RDESE were dB. The method of IASE is presented in Figure 1.

Except for the process of extracting the eddy current vector extracted, there were another three steps of the RDESE algorithm:

(1) Establish a discrete random variable. In this algorithm, the circle from the center of the specimen was divided into a series of annuluses with the same thickness. In this study, the radius of the circle was 15 mm and the thickness of the annuluses was 0.5 mm, so the number of the events in the established discrete random variable was 30. The most inside annulus was defined as the first event and the sequence number of the event gradually increased as of the radius of the annuluses increased.
(2) Calculate the radial direction energy spectrum. In this step, if an eddy current vector $\left(J_{x},\;J_{y}\right)$ was extracted at point (x,y) and point (x,y) was in the *i*-th annulus, the energy of the eddy current $J^{2}=J_{x}^{2}+J_{x}^{2}$ was added to the probability of the *i*-th event $p_{r}\left(i\right)$. Then, the $p_{r}\left(i\right)$ was normalized. This step can be expressed as
  (3) $$p_{r}\left(i\right)=\frac{\sum J_{0.5\left(i-1\right)<r\leq 0.5i}^{2}}{\sum J_{0\leq r\leq 15}^{2}}$$
  where $r=\sqrt{x^{2}+y^{2}}$ is the distance between the eddy current extracted point and the center of the specimen.
(3) Use the equation of the Shannon information entropy to calculate the index RDESE $H_{\theta }$. This step can be expressed as
  (4) $$H_{r}=-\overset{30}{\underset{i=1}{\sum }}p_{r}\left(i\right)logp_{r}\left(i\right)$$

**Figure 2:**
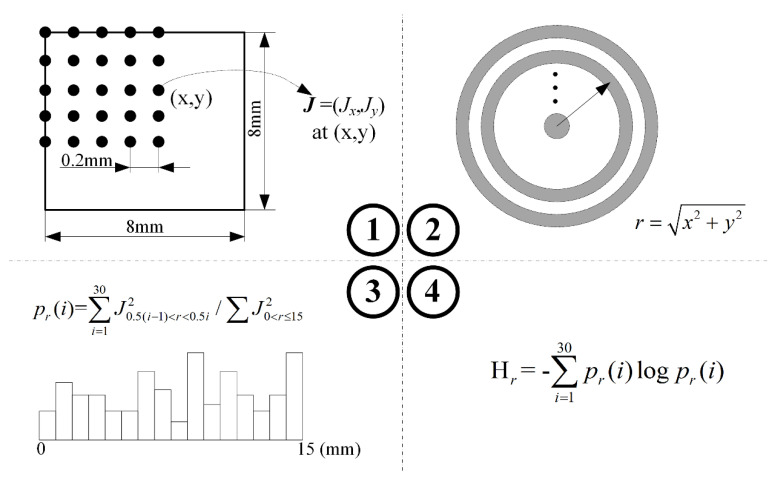
The four steps to calculate the index of the radial direction energy spectrum entropy (RDESE).

The method of the RDESE algorithm is shown in Figure 2.

## 3. Result and Discussion

**Figure 3:**
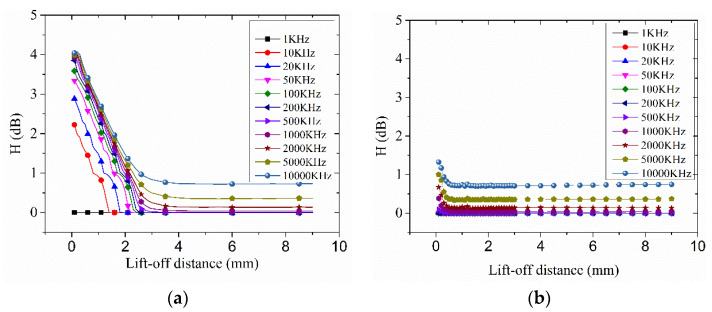
The calculated result by IASE: (**a**) Koch; (**b**) circle.

**Figure 4:**
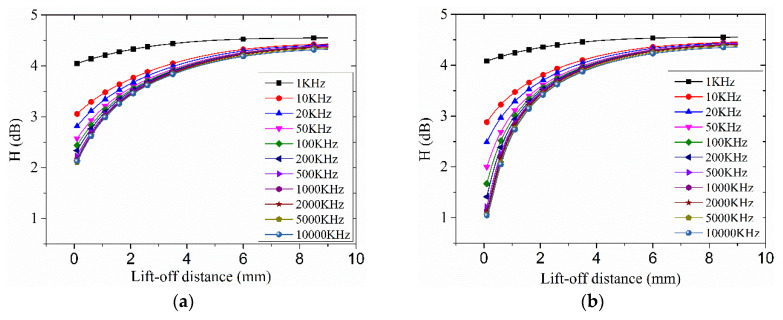
The calculated result by RDESE: (**a**) Koch; (**b**) circle.

Figure 3 and Figure 4 show the analysis result of the eddy current distribution induced by a second order Koch curve and a circle using the two kinds of the proposed algorithms.

### 3.1. The Analysis of IASE

To study the similarity between the eddy current distribution and the shape of the exciting coil of an eddy current sensor, the eddy current distributions induced by a second order Koch curve and a circular exciting coil with the different lift-off distance and exciting frequency were analyzed by RDESE algorithm mentioned before, as shown in Figure 3.

Figure 3a represents the IASE of the Koch curve exciting coil feed with the different frequency alternative current as the lift-off distance increased. For the IASE algorithm, the sampling space was the intersection angle between the perpendicular direction of the position vector of the eddy current data extraction point and the direction of the eddy current, so the larger the RDESE, the more similarity there is between the exciting coil and its eddy current distribution. In this result, when the exciting frequency was 1 KHz, the IASE was almost about 0 dB. However, when the exciting frequency was greater than or equal to 10 KHz, the IASE of every exciting frequency dropped from an initial high value at the beginning of the increase of the lift-off distance, then the IASE of every exciting frequency reached and almost remained a final constant value. The final constant value of 10,000 KHz was about 0.74 dB, which was the largest constant value among the constant values of all exciting frequencies. 

If the lift-off distance was fixed, the IASE that varied with frequency was different between the beginning of the decreasing segment and the final constant value segment of the IASE. At the beginning of the decreasing segment, the IASE increased as the exciting frequency increased but the change of IASE decreased as the exciting frequency increased. Then, in the constant value segment, when the exciting frequency was less than 1000 KHz, the constant of the IASE was about 0 dB; when the exciting frequency was larger than 1000 KHz, the constant value increased as the exciting frequency increased. From the above, there are two ways to increase the value of the IASE: reducing lift-off distance and increasing exciting frequency.

For the circular exciting coil, the variation of the IASE was much different to that of the Koch exciting coil. The line of the IASE of the circular exciting coil also had a decreasing segment and a constant value segment but the decreasing segment was shorter than that of the Koch exciting coil and when the lift-off distance was larger than 0.5 mm, all of the IASE of the circle remained almost constant as the lift-off distance varied. For a certain frequency and a certain lift-off distance, the value of the IASE of the Koch coil in its decreasing segment was larger than that of the circle. Thus, the advantage of the Koch coil as an eddy current senor exciting coil over the circular coil was limited in the small lift-off distance and high exciting frequency.

According to the analysis of the IASE of the second order Koch curve and the circular exciting coils, the IASE would decrease as the lift-off distance increased or the exciting frequency decreased. Thus, with the lift-off distance increasing and the exciting frequency decreasing, the similarity between the Koch curve exciting coil and its eddy current distribution would weaken and the information of the Koch curve in its eddy current distribution died out. With the exciting frequency increasing and the lift-off distance increasing, the eddy current distribution of the Koch curve exciting coil approached that of the circular exciting coil.

### 3.2. The Analysis of RDESE

The index of the RDESE was proposed to evaluate the degree of the eddy current distribution concentration. The eddy current distribution in the specimen under the exciting coil is stronger than that at other places of the specimen but the concentration of the eddy current distribution may change with the lift-off distance, so the index of the RDESE was proposed. If the value of the RDESE was larger, the eddy current distribution was less concentrated and vice versa.

The evaluation result of the RDESE induced by two kinds of the exciting coils was shown in Figure 4. With the exciting frequency decreasing and the lift-off distance increasing, the RDESE of the two kinds of the exciting coils increased and moved eventually towards a constant value, so the concentration of the eddy current distribution weakened. 

For the circular exciting coil with the 10,000 KHz exciting frequency alternative current, when the lift-off distance was 0.1 mm, the RDESE was about 1.05 dB but when the lift-off distance was 9 mm, the RDESE was 4.36 dB, which was 4.15 times than that of 0.1 mm lift-off distance. For the Koch exciting coil with the 10,000 KHz exciting frequency, when the lift-off distance was 0.1 mm, the RDESE was 2.13 dB but when the lift-off distance was 9 mm, the RDESE was 4.32 dB, which was 2.98 times than that of the 0.1 mm lift-off distance. 

For each frequency except 1 KHz, the variation trends of each exciting coil of each exciting frequency and lift-off distance were similar to each other. For each exciting frequency and exciting coil, the RDESE reached to constant values which ranged between 4.20–4.60 dB. However, for the 1 KHz exciting frequency, the RDESE of each lift-off distance were larger than 4.00 dB, which was very close to the eventual value above-mentioned.

## 4. Conclusions

In this paper, the index of IASE was proposed to evaluate the degree of similarity of the exciting coil and its inducing eddy current distribution. The index of the RDESE was proposed to evaluate the concentration of the eddy current distribution in the specimen under the exciting coil. The eddy current distributions of a second order Koch curve and the same size circular exciting coil were simulated with different exciting frequencies and lift-off distances. Then, the eddy current vector data were extracted and analyzed by way of the two proposed novel indices.

Thus, the effect of the increase in the lift-off distance between the exciting coil and the surface of the specimen may include the following characteristics:

(1) The magnitude of the eddy current will decrease, which leads to the lift-off noise in the output signal of the eddy current sensors [1]. This phenomenon is the traditional lift-off effect.
(2) With the increase in the lift-off distance, the information of the exciting coil in the eddy current distribution gradually decreased. This phenomenon can be concluded by the proposed index of the IASE above-mentioned.
(3) With the increase in the lift-off distance, the degree of the concentration of the eddy current distribution in the specimen under the exciting coils died down. This phenomenon can be concluded by the proposed index of the RDESE above-mentioned.

In the future, the lift-off effect of the Koch curve exciting coil eddy current sensor will be studied in an experiment.
